# Identification of the Plasma Metabolomics as Early Diagnostic Markers between Biliary Atresia and Neonatal Hepatitis Syndrome

**DOI:** 10.1371/journal.pone.0085694

**Published:** 2014-01-08

**Authors:** Dongying Zhao, Lianshu Han, Zhengjuan He, Jun Zhang, Yongjun Zhang

**Affiliations:** 1 XinHua Hospital, Shanghai Jiaotong University School of Medicine, Shanghai, China; 2 MOE and Shanghai Key Laboratory of Children’s Environmental Health, Shanghai, China; Clermont Université, France

## Abstract

Early detection is the most effective way to improve the clinical outcome of biliary atresia (BA). Emerging metabolomics provides a powerful platform for discovering novel biomarkers and biochemical pathways to improve early diagnosis. The aim of this study is to find the potential biomarkers to distinguish BA from neonatal hepatitis syndrome (NHS) by using a metabolomics method. We comprehensively analyzed the serum metabolites in a total of 124 blood samples from patients with BA or neonatal hepatitis syndrome (NHS) and from normal individuals using advanced metabolomic approaches, and found that the levels of glutarylcarnitine (C5DC) significantly increased in the BA group while the levels of threonine (Thr) significantly rose in the NHS group comparing with the other groups. The levels of glutamic acid (Glu) in the BA group were significantly elevated compared to those in the NHS group, but still lower than the hyperbilirubinemia and normal controls. The levels of propionyl carnitine (C3), isovaleryl carnitine (C5) and glutamine (Gln) were reduced in the BA group compared to those in the NHS group, but still higher than the hyperbilirubinemia and normal controls. This study demonstrates the possibility of metabolomics as non-invasive biomarkers for the early detection of BA and also provides new insight into pathophysiologic mechanisms for BA.

## Introduction

Biliary atresia (BA) is one of the most serious hepatobiliary diseases during infantile period, accounting for about 75% of liver transplantations in children younger than 2 years [1]. The reported incidence of BA varies from 5/100,000 to 32/100,000 live births [2]. The etiology and pathogenesis of BA are largely unknown. Virus infection and autoimmune disorders are the two major theoretic mechanisms [3]–[6]. Since the Kasai procedure and liver transplantation have developed over the years, BA can be treated or cured. However, the better outcomes for infants depend on early diagnosis and timely Kasai portoenterostomy. It is generally known that children operated before day 60 achieve biliary drainage and stabilization in up to 80% of cases, while in patients operated only after day 90, the success rate decreases to 20% [7]. Therefore it is extremely important to differentiate BA from other causes of neonatal cholestatic jaundice promptly and accurately. However, besides BA, neonatal hepatitis syndrome (NHS) is another major cause of cholestatic jaundice in infants. The clinical manifestations and liver function test between the two diseases are too similar to distinguish. Up to date, there is still no accuracy investigation to diagnose BA. For example, the abdominal ultrasound of BA has a high specificity but low sensitivity, while the hepatobiliary scintigraphy (HBS) has a high sensitivity but low specificity [8], [9]. The “gold standard” of diagnosing BA is liver biopsy, which is invasive. Therefore, more sensitive and specific biomarkers for the early diagnosis of BA are needed.

Metabonomics is concerned with the measurement of global sets of low molecular weight metabolites, which can be regarded as important indicators of various states of disease. Through analyzing and verifying the specific biomarkers of a disease, metabolomics enables us to better understand pathological processes, and substance metabolic pathways. Compared with traditional diagnostic methods, even small changes of metabolites can help to detect early pathologic changes more sensitively [10], [11]. Its utility has been demonstrated by the identification of new biomarkers for Hepatocellular Carcinoma, Kidney Injury, and Alzheimer’s disease [11]–[13].

Today, the improved efficiency and accuracy of metabolomic biomarkers and early diagnostic technologies have been increasingly used in a clinic setting. By applying advanced analytical and statistical tools, the “metabolome” is mined for biomarkers that are associated with the state of BA. It may help to understand the mechanism of BA occurrence and progression on the metabolic level and provide information for the identification of early and differential marker metabolites for BA. Thus, the aim of this study was to evaluate the usefulness of the metabolic alterations as a rapid metabolomics screening technique for BA. As such, the identification of serum biomarkers may provide useful information for early diagnosis of BA, and effectively distinguish BA from other diseases such as NHS and hyperbilirubinemia.

## Methods

### Ethics and Clinical Populations

This study was a prospectively randomized controlled trial, whose design has been approved by the Ethical Committees of the Xinhua Hospital affiliated to Shanghai Jiaotong University School of Medicine. All parents or legal guardians signed an agreement after being disclosed to necessary information on this study.

From January 2008 to December 2011, a total of 61 cholestasis infants were included in the study and 32 hyperbilirubinemia infants and 31 normal infants as control. For all patients, serum alanine transaminase (ALT), aspartate transaminase (AST), total bilirubin (TB), direct bilirubin (DB), γ-glutamyl transpeptidase (γ-GT), total bile acid (TBA) and alkaline phosphatase (ALP) were measured. Four dried blood spots samples were obtained from infants by heel prick and spotted on filter paper [S&S 903# (CFH)] at the patients’ first presentation to hospital and then stored at −20°C.

Cholestasis infants were defined as conjugated bilirubin more than 20% of the total bilirubin when the total bilirubin is 85 µmol/L or more and 17 µmol/L or more when the total bilirubin is 85 µmol/L or less [14]. First, history taking and physical examination of all patients were conducted carefully. And then they underwent differential diagnosis and etiologic work-up of cholestasis, including a complete blood count, virus biomarkers, serum-concentration of α_1_-antitrypsin, blood tandem mass spectrometry and urine gas chromatography inspection as well as magnetic resonance cholangiopancreatography, abdominal ultrasound and HBS. In addition, specific biochemical tests were done as needed. For all, there were 45 cases suspicious for BA underwent intraoperative cholangiography and liver biopsy. And all of them had a 3 to 6 month clinical follow-up. Exclusion criteria: 1. Cholestasis infants with any family history such as progressive familial intrahepatic cholestasis, (PFIC), α_1_-antitrypsin deficiency; 2. Cholestasis infants with other obvious accompany manifestation like endocrine disorders, chromosomal disorders and Alagille’s syndrome. 3. Cholestasis infants with any amino, organic, and fatty acid disorders such as neonatal cholestasis induced by citrin deficiency (NICCD), tyrosinemia. Diagnostic criteria: 1.Hyperbilirubinemia group: Infants had a serum bilirubin up to 221 µmol/L or clinical jaundice appearing greater than 14 days of life with a conjugated bilirubin less than 20% of the total bilirubin. 2. Biliary atresia group: the confirmed diagnosis of BA was based on laparoscopy with surgical cholangiography and liver biopsy. The gallbladders or the remnants of gallbladders were found, but extrahepatic biliary duct could not be detected during the operation. And liver biopsy showed the typical histopathologic features, including bile duct proliferation, bile plugs, giant cell transformation, canalicular and cellular bile stasis and periportal fibrosis [15]. At last, 30 patients were identified as BA. 3. Neonatal hepatitis syndrome group: There were 15 infants who had undergone laparoscopy with surgical cholangiography and liver biopsy. During the operation all of them showed biliary tree and the giant cell transformation, inflammatory cell infiltration, and lobular disorganization on liver biopsy. They were finally diagnosed as NHS. Additionally, 16 patients recovered from jaundice and had the normalization of laboratory values during the clinical follow-up for about 3–6 months. So it was unnecessary to perform an intraoperative cholangiography. We confirmed these patients as idiopathic neonatal hepatitis. Finally, 30 cases of BA and 31 cases of NHS were enrolled in this study.

### Samples Preparation

At the time of LC-MS/MS analysis, the frozen blood spots samples were thawed in normal atmospheric temperature. According to the previous report [16], three millimeter discs were punched and incubated in the methanolic internal standard solution (100 µL) for 20 min. The internal standards of amino acids and acylcarnitine were purchased from Cambridge Isotope Labs, USA. The concentrations of the deuterium-labeled internal standards per liter of methanol were as follows: [^15^N,2-^13^C_1_]-glycine ([N1]-[C1]-Gly), 12.5 µmol/L; [^2^H_4_]- alanine([D4]-Ala), 2.5 µmol/L; [^2^H_8_]- valine ([D8]-Val), 2.5 µmol/L; [^2^H_3_]-leucine ([D3]-Leu), 2.5 µmol/L; [^2^H_3_]-methionine ([D3]-Met), 2.5 µmol/L; [^2^H_5_]-phenylalanine([D5]-Phe),2.5 µmol/L; [^13^C_6_]-tyrosine([C6]-Tyr), 2.5 µmol/L; [^2^H_3_]-aspartic acid ([D3]-Asp), 2.5 µmol/L; [^2^H_3_]-glutamic acid ([D3]-Glu), 2.5 µmol/L; [^2^H_2_]-ornithine ([D2]-Orn), 2.5 µmol/L; [^2^H_2_]-citrulline ([D2]-Cit), 2.5 µmol/L; [^2^H_4_-^13^C_1_]-arginine ([D4]-[C1]- Arg), 2.5 µmol/L; [^2^H_9_]-carnitine([D9]- C0), 0.76 µmol/L; [^2^H_3_]-acetylcarnitine([D3]-C2), 0.19 µmol/L; [^2^H_3_]-propionylcarnitine ([D3]-C3), 0.04 µmol/L; [^2^H_3_]-butyrylcarnitine([D3]-C4), 0.04 µmol/L; [^2^H_9_]-isovalerylcarnitine([D9]-C5), 0.04 µmol/L; [^2^H_3_]-octanoylcarnitine ([D3]-C8), 0.19 µmol/L; [^2^H_9_]-myristoylcarnitine([D9]-C14), 0.04 µmol/L; [^2^H_3_]-palmitoylcarnitine ([D3]-C16), 0.08 µmol/L. The diluted sample was then manually transferred to a 96 well polypropylene microtiter plate (membrane pore size 0.2/0.45 µm, Millipore Corporation, USA) and dried by a hot air blower (Dri-Block DB-30, Techne, Britain) at 55°C. Each diluted sample was placed in 65°C forced air oven for 15 min by the addition of 60 µl of n-butanol hydrochloride (3 mol/L), covered with a thin Teflon sheet. After the plate was removed from the oven, the hot air blower removed the butanol hydrochloride. The butanol-derivatized samples were reconstituted with 100 µL of acetonitrile and water (80∶20 by volume), and each plate was covered with aluminum foil. The samples were then ready for MS/MS analysis.

### LC-MS/MS Measurements

**Figure 1:**
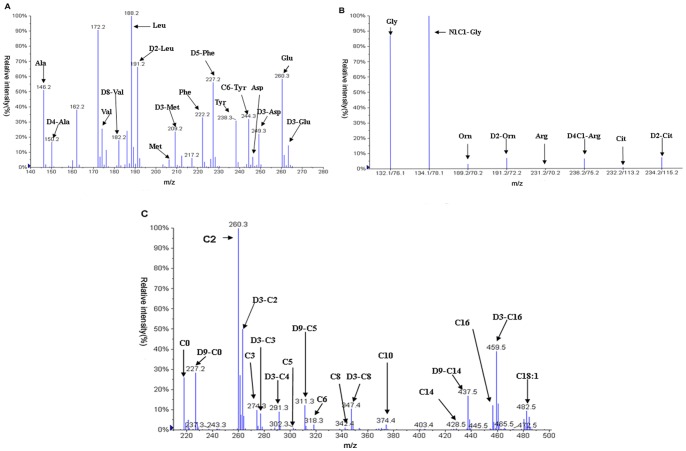
A typical LC-MS/MS spectrum of blood spots from patients. Blood samples were prepared and mass spectrometric analysis of individual sample was performed as described in “Methods” in details. Panel A shows a mass spectrum was acquired in the neutral loss scan mode. The scan was range from m/z 140 to m/z 280. It can detect most of the amino acids. Panel B shows a mass spectrum was acquired in the multiple reaction monitor mode. It was mainly used to detect Gly, Orn, Arg, Cit and its internal standard. Panel C shows a mass spectrum was acquired in precursor ion scanning mode. It used to detect the acylcarnitine in the sample.

An API Mass spectrometer 2000 (Applied Biosystems, USA) coupled with a HPLC system of Agilent 1000 (Agilent Technologies, USA) was used to detect amino acids and acylcarnitine in the samples. Twenty microliters of prepared sample and solvent were introduced by autosampler into MS1 of the API Mass spectrometer 2000. Mobile phase used 80% acetonitrile: water and quaternary pump were used to set flow speed: 140 µl/min, 0.2 min→30 µl/min,1 min→300 µl/min, 0.2 min. The obtained spectra of amino acids were analyzed with neutral loss scan (neutral loss fragment butyl formate, m/z102) and multiple reaction monitoring. Neutral loss scan range of m/z 140 to m/z 280 and multiple reaction monitoring were test for Gly, Orn, Arg, Cit and its internal standard. The obtained spectrum of acylcarnitine in every sample was using precursor ion scanning mode. The precursor ion scan range from m/z 210 to m/z 502 and the product ion fragment was m/z 85. Hexanoylearnitine (C6) has no internal standards. As the mass to charge ratio of butyl-esterified C6 was 316.3, we detected it with D9-C5 quantitatively. The scan time of all the amino acids and acylcarnitine was set to 1.4 minutes and the each test sample would be detected with four minutes. The mass to charge ratio of all the amino acid and acylcarnitine was shown in Table 1 and the spectra were seen in Fig. 1.

**Table 1:** The scanning mass-to-charge ratio of amino acids and acyl carnitine.

Name	mass-to-chargeratio	Name	mass-to-charge ratio
Ala	m/z 146.1	Cit	m/z 232.1/m/z 113.1
D4-Ala	m/z 150.1	D2-Cit	m/z 234.2/m/z 115.2
Val	m/z 174.2	C0	m/z 218.2
D8-Val	m/z 182.2	D9-C0	m/z 227.2
Met	m/z 206.1	C2	m/z 260.2
D3-Met	m/z 209.1	D3-C2	m/z 263.2
Phe	m/z 222.2	C3	m/z 274.2
D5-Phe	m/z 227.2	D3-C3	m/z 277.2
Tyr	m/z 238.1	C4	m/z 288.2
C6-Tyr	m/z 244.1	D3-C4	m/z 291.2
Asp	m/z 246.2	C5	m/z 302.2
D3-Asp	m/z 249.2	D9-C5	m/z 311.2
Gly	m/z 132.1/m/z 76.1	C8	m/z 344.3
N1-C1-Gly	m/z 134.1/m/z 78.1	D3-C8	m/z 347.3
Orn	m/z 189.2/m/z 70.2	C14	m/z 428.4
D2-Orn	m/z 191.2/m/z 72.2	D9 -C14	m/z 437.4
Arg	m/z 231.2/m/z 70.2	C 16	m/z 456.4
D4-C1-Arg	m/z 236.2/m/z 75.1	D3-C16	m/z 469.4

### Data Processing

The data were analyzed by Chem View b5, (U.S. Applied Biosystems), which was in-house analytical software in API 2000 MS. Based on a variety of amino acid ester and acylcarnitine and its isotopic ion peak intensity of the internal standard, this software enables peak identification and quantification using an in-house metabolite library. The markers were expressed as “concentration” or as a “ratio” and were exported in EXCEL format. The overall resultant data included 17 amino acids (Alanine [Ala], Aspartic acid [Asp], Glutamic acid [Glu], Methionine [Met], Phenylalanine [Phe], Tyrosine [Tyr], Leucine [Leu], Tryptophane [Trp], Valine [Val], Argnine [Arg], Citrulline [Cit], Glycine [Gly], Ornithine [Orn], Glutamine [Gln], Histidine [His], Serine [Ser], Threonine [Thr]) and 30 acylcarnitine concentrations (Free carnitine [C0], Acetyl carnitine [C2], Propionyl carnitine [C3], Malonyl carnitine [C3DC], Butyrylcholinesterase carnitine [C4], 3-hydroxybutyrate acyl carnitine [C4-OH], Succinic acid carnitine [C4DC], Isovaleryl carnitine [C5], Senecioyloxy carnitine [C5:1], 3-hydroxy-isovaleryl-carnitine [C5-OH], Glutaryl carnitine [C5DC], Hexanoyl carnitine [C6], Hexene acyl carnitine [C6:1], Adipic acid carnitine [C6DC], Octanoyl carnitine [C8], Octene carnitine [C8:1], Sim two acyl carnitine [C8DC], Kwai acyl carnitine [C10], Aoi enoyl-carnitine [C10:1], Lauroylcarnitine [C12], Myrcene carnitine [C12:1], Myristoyl carnitine [C14], Nutmeg enoyl-carnitine [C14:1], 3-hydroxy-myristoyl-carnitine [C14-OH], Palmitoyl carnitine [C16], Palm enoyl-carnitine [C16:1], 3-hydroxy-palmitoyl carnitine [C16-OH], Octadecadienoic acid carnitine [C18], Carnitine octadecene [C18:1], 3-hydroxy-octadecadienoic acid carnitine [C18-OH]) as well as 7 amino acid ratios (Arg/Orn, Cit/Arg, Orn/Cit, Met/Phe, Leu/Phe, Phe/Tyr, Gly/Phe) and 8 acylcarnitine ratios (C3/C0, C3/C2, C4/C2, C5/C2, C5-OH/C2, C5DC/C8, C8/C2, C14:1/C8:1).

### Statistical Analysis

Orthogonal partial least squares discriminant analysis (OPLS-DA) was applied to analyze LC-MS spectral data by SIMCA-P+ (v12.0 version, Umetrics, Umeå, Sweden). The number of components for OPLS-DA models was auto-fitted using a cross validation. OPLS-DA, a supervised pattern recognition (PR) method, was conducted to maximize the difference between the BA and other groups. The variable importance for projection (VIP) score for each component was derived from the OPLS-DA analysis and served as an indication of contribution to the OPLS model. The quality of the models was assessed by a 10-fold cross-validation method. The obtained parameters R^2^ stands for the total explained variation of the model and describes how well the derived model fit the data, and Q^2^ represents the predictability of the model and provides a measure of the model quality. According to a greater-than-one threshold of VIP in the OPLS-DA model, a number of variables are responsible for the differences in the metabolic profiling between BA, INH, hyperbilirubinemia and normal groups. Variables that were normally distributed and had homogeneous variance were then analyzed by one-way ANOVA with a LSD test from SPSS Statistics 12.0 (SPSS, Chicago, IL, USA). We examined if different biomarker candidates obtained from the OPLS-DA models were statistically significant among the four groups at the univariate level. A p-value <0.05 (confidence level 95%) was considered statistically significant. Besides, other distributed data were also analyzed using one-way ANOVA followed by a LSD test by SPSS.

## Results

### Demographic Characteristics

There were a total of 124 patients including 67 boys and 57 girls with a mean age of 39.8±26.9 days and a mean weight of 4.3±1.1 kg. These infants were divided into four groups as described above. Table 2 shows clinical data including the patients’ demographic information and serum liver function values. There was no apparent difference in the age of presentation, sex distribution and the liver function parameters including TB, DB, ALT, AST, TBA and ALP between NHS and BA groups. However, the γ-glutamyl transpeptidase (γ-GT) value was extremely higher in BA group than other groups.

**Table 2:** Comparison of clinical data among four groups.

Parameters	BA	NHS	Hyperbilirubinemia	Normal	Significance
N	30	31	32	31	
Male	17	21	14	15	N.S.1
Female	13	10	18	16	
Age at presentation(d)					
mean	58.5	54.8	11.1a	46.5	<0.052
sd	27.5	21.8	7.8	14.8	
range	25–120	25–150	3–38	19–81	
Weight at presentation (kg)					
mean	4.6	4.5	3.4 a	4.4	<0.052
sd	1.0	1.3	0.6	0.8	
TB(µmol/l)					
mean	163.0b	137.1b	283.2a	26.6	<0.052
sd	57.5	85.8	54.4	25.9	
DB(µmol/l)					
mean	112.9b	97.5b	19.8	6.4	<0.052
sd	39.0	64.1	16.0	4.8	
ALT(U/L)					
mean	150.8b	205.2b	16.2	23.1	<0.052
sd	87.6	122.9	7.0	9.8	
AST(U/L)					
mean	241.1b	286.0b	50.7	41.3	<0.052
sd	125.5	250.4	30.9	18.9	
γ-GT (U/L)					
mean	552.5c	277.0b	137.6	81.5	<0.052
sd	353.9	185.7	61.9	69.6	
TBA(µmol/l)					
mean	116.4b	139.5b	12.7	16.1	<0.052
sd	43.3	99.1	10.8	11.7	
ALP(U/L)					
mean	507.2b	567.9b	193.9	70.8	<0.052
sd	253. 5	224.5	63.7	12.7	

^1^χ2-test.

2 one-way ANOVA followed by a LSD Test.

a Comparison vs normal group p<0.05.

b Comparison vs hyperbilirubinemia and normal group p<0.05.

c Comparison vs NHS group, hyperbilirubinemia and normal group p<0.05.

### Discovery and Identification of Metabolic Biomarkers

**Figure 2:**
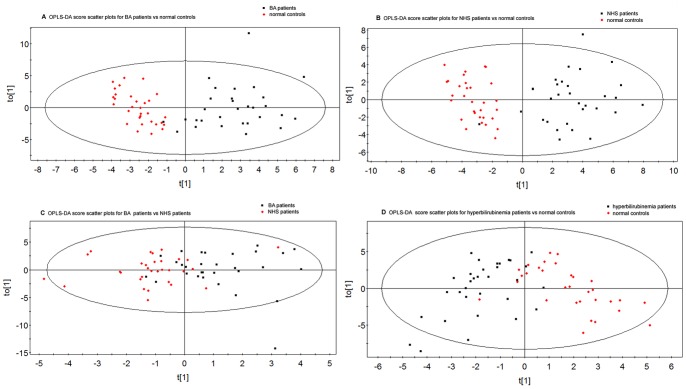
OPLS-DA score scatter plots obtained from of LCMS analysis of samples. A. OPLS-DA score plots of BA and normal; B. OPLS-DA score plots of with NHS and normal; C. OPLS-DA score plots of BA and NHS; D. OPLS-DA score plots of hyperbilirubinemia and normal. The fig. showing that the two populations are well separated respectively.

The LC-MS variables obtained from all subjects were used to construct an OPLS-DA model. In those four groups, we analyzed two sets of pair-wise data to identify the metabolic biomarkers to discriminate the BA and NHS groups. Fig. 2 show a clear separation between BA and normal group, NHS and normal group, BA and NHS group, hyperbilirubinemia and normal group. Table 3 indicates the performance parameters of the four models, suggesting that these models were reliable and highly predictive.

**Table 3:** Summary of statistical values of OPLS-DA with different scaling methods for data obtained from LC-MS analyses^a^.

Comparison model	N	R^2^X (cum)	R^2^Y (cum)	Q^2^ (cum)
BA vs normal group	61	0.486	0.769	0.619
NHS vs normal group	62	0.382	0.787	0.704
BA vs NHS	61	0.269	0.410	0.242
hyperbilirubinemia vsnormal group	63	0.321	0.601	0.429

a Including the different cumulated modeled variations in X [R^2^X(cum)] and Y [R^2^Y(cum)] matrix on spectral datasets and predictability of the model [Q^2^(cum)].

**Figure 3:**
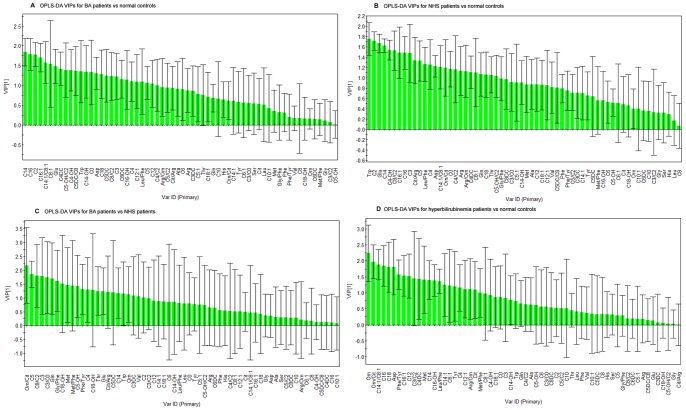
The amino acid and acylcarnitine with a VIP value above 1.0 were identified as markers to discriminate the two comparing group. The Variables were aligned in descending order of their VIP values. From model A and model B, we can obtain the identified metabolites of BA and NHS. From model C, we can verify these metabolites obtained above can distinguish BA from NHS. From model D, we can exclude the interference of metabolites in hyperbilirubinemia infants.

The VIP score for each compared model was generated for each OPLS-DA analysis. A VIP score >1.0 implied that the metabolite contributed significantly towards the differentiation of the two compared groups, shown in Fig. 3.

As shown in Fig. 3, from model A and model B, we obtained the specific metabolites of BA and NHS group, compared with the normal group respectively. From model C, we verified whether these metabolites obtained above could distinguish BA from NHS. And from model D, we excluded the interference of metabolites in hyperbilirubinemia infants. Finally, according to the criterion for VIP statistics (VIP>1), we obtained a total of nine metabolites for their most contribution in discriminating profiles between BA and NHS cohorts. Their VIPs were shown in Table 4. Then One-way analysis of variance (ANOVA) test by LSD was used to examine the statistical significance of these metabolites among the four groups. At last, six of which were detected with significant differences. (Table 5). The results suggest that the levels of C5DC significantly increased in the BA group, while in the NHS group, the levels of Thr significantly rose. The levels of Glu were significantly elevated in the BA group compared to those in the NHS group, but still lower than the hyperbilirubinemia and normal controls. The levels of C3, C5 and Gln in BA were significantly reduced compared to those in the NHS group, but still higher than those in the hyperbilirubinemia and normal controls.

**Table 4:** VIP value of the potential metabolites in the differences of NHS and BA^a^.

Metabolites	BA vs normal group	NHS vs normal group
	VIP	VIP
C5DC	1.3	<1.0
Cit/Arg	<1.0	1.4
Gln	<1.0	1.1
Thr	<1.0	1.1
Glu	1.5	1.7
Trp	1.4	1.7
C2	1.7	1.7
C3	1.2	1.5
C5	1.1	1.3

a VIP: The variable importance for projection.

**Table 5:** Comparison of the potential metabolites in different groups by LSD post hoc analysis.

Metabolites	BA	NHS	Hyperbilirubinemia	Normal
C5DC (µmol/l)				
mean	0.06^a^	0.05	0.04	0.04
sd	0.02	0.02	0.01	0.01
C2(µmol/l)				
mean	22.94^b^	26.54^b^	12.70	11.61
sd	10.24	9.14	5.04	4.17
C3 (µmol/l)				
mean	2.68^a^	3.68^b^	1.17	1.49
sd	1.64	1.67	0.66	1.00
C5 (µmol/l)				
mean	0.12^a^	0.17^b^	0.08	0.09
sd	0.06	0.08	0.03	0.03
Cit/Arg				
mean	3.13^b^	2.23^b^	4.66	4.66
sd	2.67	1.20	2.13	2.06
Gln (µmol/l)				
mean	9.50^a^	12.33^b^	5.41	7.10
sd	4.44	3.93	5.61	5.54
Thr (µmol/l)				
mean	59.46	73.07^c^	50.96	46.61
sd	32.22	35.45	17.49	14.95
Glu (µmol/l)				
mean	204.62^a^	171.21^b^	279.32	282.56
sd	83.53	52.35	55.11	52.00
Trp (µmol/l)				
mean	43.47^b^	35.26^b^	68.37	63.93
sd	26.82	14.54	13.71	12.03

a Have a significant difference at P<0.05 compared to NHS group, hyperbilirubinemia and normal group.

b Have a significant difference at P<0.05 compared to hyperbilirubinemia and normal group.

c Have a significant difference at P<0.05 compared to BA group, hyperbilirubinemia and normal group.

## Discussion

Since the Kasai hepatoportoenterostomy has been evolving over the years, more and more studies have demonstrated that the earlier age at Kasai was a predictor of improved native liver survival and overall survival of BA [17], [18]. But it is still difficult to differentiate BA from other causes of neonatal cholestatic jaundice especially the NHS. Because of limitations in conventional approaches, several noninvasive tests have been developed to facilitate such a diagnosis. With recent advances in metabonomics, serum metabolites could be used as a novel diagnostic indicator for disease [19].

Accruing evidence suggests that metabolic profiles are useful for identification of diagnostic biomarkers in BA. Zhou and Mushtaq have suggested that bile acids elevated in biliary atresia by comparing with other forms of cholestatic hepatobiliary diseases or neonatal jaundice group basing on tandem mass spectrometry [20], [21]. In the future, we also intend to further investigate the metabolomics of amino acids and acylcarnitine profiles of BA. In this study, we attempted to identify sensitive and specific metabolites to distinguish BA form NHS based on LC/MS, which does not require the time-consuming derivatization procedure and is suitable for analysis of the metabolites compounds [22]. Because the symptoms of BA initially are indistinguishable from neonatal hyperbilirubinemia and later other symptoms like clay colored stools, dark urine, and large hardened liver are indistinguishable from NHS. We set both hyperbilirubinemia and NHS as the study groups.

In the previous studies, there were different amino acid and acylcarnitine metabolite profiles corresponding to the different forms of liver disease [11], [23]–[27]. However there were only a few studies on BA using LC-MS-based metabonomic methods to investigate the acylcarnitine profiles. As generally known, carnitine plays an important role in fatty acid oxidation and energy production. It helps long-chain fatty acids to enter into the mitochondria for the purpose of β-oxidation and regulating the proportion and coenzyme A and acylcoenzyme A. The carnitine bound with acyl-CoA to form acylcarnitine, which help the elimination of organic acid. Animal experiments indicate that biliary export was a major route for acylcarnitine clearance and long-chain acyl-carnitine was hardly excreted through bile duct [28], [29]. So short-chain carnitine are the mainly excretion of carnitine in the bile duct. Selimoglu MA et al reported that plasma carnitine levels were significantly increased in children with biliary atresia [23]. In this study, we also found that there was a set of short-chain carnitine metabolites elevating in the BA patients. Among them, the C5DC concentrations in BA were higher than the other three groups, and C2, C5 concentrations in BA are statistically lower than NHS group but still higher than hyperbilirubinemia and normal controls. The elevated glutarylcarnitine (C5DC) suggested the deficient or nonfunctional of glutaryl-CoA dehydrogenase. And this was usually seen in the Glutaric aciduria type I disorder, acting as an important neurological dysfunction resembling an episode of encephalitis with movement disorders such as dystonia and/or dyskinesia [30]. However, in our study, the BA patients did not show such clinical onset, so the further mechanisms that lead to the pathophysiological changes are to be studied.

As the liver plays a central role in amino acid metabolism, it is of great importance to investigate the metabolite profiles in BA and NHS. From the liver function tests, we found that there was no significantly different elevation of serum ALT and AST in BA and NHS patients. However, there was a set of significantly different amino acids concentrations between BA and NHS.

Gln significantly increased while Glu significantly decreased in the serum of NHS patients compared with BA patients in our study. Coincidentally, these amino acids participate in the urea cycle and these changes suggest impairment in the urea cycle. Urea cycle describes the conversion reactions of ammonia into urea, which help the toxic ammonia removed from the body. A lot of reports on proteomic analysis of urea cycle revealed the regulation of urea cycle enzymes had dysfunction in the liver injury such as glutamine synthetase, glutamate dehydrogenase [31], [32]. As a result, the decreased levels of Glu and increased levels of Gln were detected in serum of NHS patients. Besides, higher levels of Thr were also found in the NHS infants, presumably because of the activity of threonine-degrading enzymes decreased. Above all, these reactions mainly occur in the liver especially in mitochondria of liver cell. So this change reflects the different degree of liver cell injury. According to the previous study, the patients with hepatitis have oxidative stress, which plays a crucial role in the induction and progression of liver injury [33]. Oxidative stress produced by inflammation in the NHS infants appears to be more active than those in the BA infants and results in a more serious liver damage. Subsequently there was a different amino acids profile between the two groups.

The aim of this study was to discover new noninvasive biomarkers for BA. We quantitatively analyzed the metabolites from patients. Our results indicate that there is a metabolic profile shift of amino acid and acylcarnitine in BA from NHS. The alterations of these metabolites suggest different disturbances in metabolism between BA and NHS. They can be potentially developed into a clinically useful diagnostic tool for contributing towards an improved understanding of a disease mechanism.
